# Supplementary material for: Structure, biosynthesis, and bioactivity of nostolysamides
Source: bioRxiv. 2026 Apr 5:2026.01.31.703028. Preprint. [Version 2] doi: 10.64898/2026.01.31.703028 (PMC13060382; doi:10.64898/2026.01.31.703028)
Supplement: Supplement 1 [file media-1.pdf]

Supporting Information

Structure, biosynthesis, and bioactivity of nostolysamides

Enleyona Weir,[1] Ivan Anterola,[1] and Wilfred A. van der Donk[1,*]

[1]Department of Chemistry and Howard Hughes Medical Institute, University of Illinois at Urbana-Champaign, Urbana, IL, 61822, USA

* corresponding author: vddonk@illinois.edu; 217 244 5360

Table S1: Plasmids used in this study S2
Table S2. Primers used in this study S3-S4
Table S3. gBlocks used in this study S4
Table S4. Accession number associated with the proteins used S5
Table S5. Strains associated with cluster 65 .... S5
Figure S1. LC-QTOF-ESI/MS profile of nostolysamide C S6
Figure S2. Reversed-phase HPLC traces of purified nostolysamide C S7
Figure S3. MALDI-TOF mass spectra of the NEM and DTT assay of **1** and **2** S8
Figure S4: ^{1}H NMR spectrum of nostolysamide C S9
Figure S5. Characterization of SUMO-NpuA-S4A modified by NpuM S10
Figure S6. MALDI-TOF MS of NpuA-C14A co-expressed with NpuM S11
Figure S7. MALDI-TOF MS of NpuA-C20A co-expressed with NpuM S12
Figure S8. Bioactivity assay with Compound **1** against gram-positive bacteria S13
Figure S9. Bioactivity assay with nostolysamide C against Candida species S13
Figure S10. Bioactivity assay with Compound **1** against gram–negative bacteria .S14
Figure S11. Bioactivity assay with Compound **2** against *Candida tropicalis* S14
Figure S12. Compound **1** does not target lipid II S15
Figure S13. Membrane depolarization measured by DiSC$_3$(5) assay S16
Figure S14. Sequence Similarity Network (SSN) of GNAT enzymes S17
Figure S15. A representative subset of genome neighborhoods from Cluster 1 ... S18
Figure S16. MALDI-TOF MS of the lysine mutants S19
Figure S17. Analysis of acylated product using SUMO-NpuN S20-21
Figure S18. Structural comparison of GNAT protein S22-23
Figure S19. Sequence alignments of the GNAT protein S24-25
Figure S20. Structure of pinensins S25
Figure S21. Sequence alignment of NpuM and ProcM S25
References S26

Supplementary Table S1: Plasmids used in this study

**Plasmids**		
pET-His$_6$-SUMO-NpuA_NpuM	pETduet derivative, expression His$_6$-SUMO-NpuA_NpuM, Ampicillin	This work
pRSFduet-His$_6$-NpuM	pRSFduet derivative, expression His$_6$-NpuM, kanamycin	This work
pRSFduet-His$_6$-NpuN	pRSFduet derivative, expression His$_6$-NpuN, kanamycin	This work
pET-His$_6$-SUMO-NpuA	pETduet derivative, expression His$_6$-SUMO-NpuA, Ampicillin	This work
pRSFduet-His$_6$-SUMO-NpuN	pRSFduet derivative, expression His$_6$-SUMO-NpuN, kanamycin	This work
pET-His$_6$-SUMO-NpuA_K1A	pETduet derivative, expression His$_6$-SUMO-NpuA_K1A, Ampicillin	This work
pET-His$_6$-SUMO-NpuA_K3A	pETduet derivative, expression His$_6$-SUMO-NpuA_ K3A, Ampicillin	This work
pET-His$_6$-SUMO-NpuA_K1A/K3A	pETduet derivative, expression His$_6$-SUMO-NpuA_ K1A/K3A, Ampicillin	This work
pET-His$_6$-SUMO-NpuA_C25A	pETduet derivative, expression His$_6$-SUMO-NpuA_ C25A, Ampicillin	This work
pET-His$_6$-SUMO-NpuA_S18A	pETduet derivative, expression His$_6$-SUMO-NpuA_ S18A, Ampicillin	This work
pET-His$_6$-SUMO-NpuA_S4A_NpuM	pETduet derivative, expression His$_6$-SUMO-NpuA_S4A_NpuM, Ampicillin	This work

Supplementary Table S2: Primers used in this study

Template	Primers	Nucleotide sequence (5` to 3`)	Plasmid produced
pET-28-His$_6$-NpuA-NpuM	NpuA_65_SUMO_F	gatcggatcctATGTCCCAGGAGAACC	pET-His$_6$-SUMO-NpuA
	NpuA_65_SUMO_R	gctcgaattcttaGCATTTGGAGCCCC	
pET- His$_6$-SUMO	pET-v-SUMO-F	TCCAAATGCtaagaattcgagctcggc	
	pET-v-SUMO-R	ATaggatccgatccaccaatctgttctctgt	
pET- His$_6$-SUMO-NpuA	pET-v-SUMO-NpuA-NpuM-F	tgagcggaattcgagctcggc	pET-His$_6$-SUMO-NpuA_NpuM
	pET-v-SUMO-NpuA-NpuM-R	ctagaccgttaGCATTTGGAGCCCC	
pET-28-His$_6$-NpuA-NpuM	pET-gM-SUMO-F	CAAATGCtaacggtctagcataaccccttg	
	pET-gM-SUMO-R	ctcgaattccgctcaaaaaacccctca	
pET- His$_6$-SUMO-NpuA	NpuAS4A-F	GAGGTAAAGGAAAGgctTGTCCGCTGGACACGC	pET-His$_6$-SUMO-NpuA_S4A_NpuM
	NpuAS4A-R	GCGGACAagcCTTTCCTTTACCTCCCGCAAC	
pET- His$_6$-SUMO-NpuA	NpuAC25A-F	GGGGCTCCAAAgcttaagaattcgagctcggcgc	pET-His$_6$-SUMO-NpuA_C25A
	NpuAC25A-F	agctcgaattcttaagcTTTGGAGCCCCAGCAACCAGAG	
pET- His$_6$-SUMO-NpuA	NpuAS18A-F	CCTGCGCgctGGTTGCTGGGGCTC	pET-His$_6$-SUMO-NpuA_S18A
	NpuAS18A-R	CCCAGCAACCagcGCGCAGGAAGCAAG	
NpuN-gblock	NpuN-F	GCAGGCGCGCCGAGC	pRSFduet-His$_6$-NpuN
	NpuN-R	CCCTGTAGAAATAATTTTGTTTAACTTTAATAAGGAGATATACCATGGGC	
pRSF-His$_6$	pRSF-v-NpuN-F	GGCGCGCCTGCAGGTC	
	pRSF-v-NpuN-R	CTCCTTATTAAAGTTAAACAAAATTATTTCTACAGGGGAATTGTTATCCG	
pRSF-His$_6$	pRSF-v-NpuM-F	CTGTGGGAAtaaAGCCAGGATCCGAATTC	pRSFduet-His$_6$-NpuM
	pRSF-v-NpuM-R	GCGTTCTTCCATGTGGTGATGATGGTGA	

pET-28-His$_6$-NpuA_NpuM	NpuM-F	CATCACCACATGGAAGAACGCGAAATGATCAAT	
	NpuM-R	GGATCCTGGCTttaTTCCCACAGAAGCAC	
pRSF_SUMO-TEV His$_6$	pRSF-v-SUMO_NpuN-F	ggctaatgcggccgcataatgct	pRSFduet-His$_6$-SUMO-NpuN
	pRSF-v-SUMO_NpuN-R	gttcagggtatacattccctggaagtacaggttttc	
pRSFduet-His$_6$-NpuN	NpuN-F	cctgtacttccagggaatgtatacccctgaacatt	
	NpuN-R	gttcagggtatacattccctggaagtacaggttttc	

Supplementary Table S3: gBlocks used in this study

NpuA_Tev_K1A	ggatcggatcctGAAAATTTATATTTTCAGAGCATGTCCCAGGAGAACCTGGAACAGTTTTACGTCCTCGTGCAAAACTCAGAACAATTACAAGAGCTGTTGGGCGCTACGGAAAACACAGATTCATTTAATGAACTCGCAGTGCGTTTAGGCCAGGATAATGGGTATAACTTTACCATCCAAGAAGTTGATGCTTTCGTCACCGAAAATCTGCAGAACGTTAACGCGGAACTGCGTGATGAAGAACTGGAACTGGTTGCGGGAGGTgcgGGAAAGTCGTGTCCGCTGGACACGCAATTCACGGCTTGCTTCCTGCGCTCTGGTTGCTGGGGCTCCAAATGCtaagaattcgagct
ENW_Tev_K1A_K3A	ggatcggatcctGAAAATTTATATTTTCAGAGCATGTCCCAGGAGAACCTGGAACAGTTTTACGTCCTCGTGCAAAACTCAGAACAATTACAAGAGCTGTTGGGCGCTACGGAAAACACAGATTCATTTAATGAACTCGCAGTGCGTTTAGGCCAGGATAATGGGTATAACTTTACCATCCAAGAAGTTGATGCTTTCGTCACCGAAAATCTGCAGAACGTTAACGCGGAACTGCGTGATGAAGAACTGGAACTGGTTGCGGGAGGTgctGGAGCTTCGTGTCCGCTGGACACGCAATTCACGGCTTGCTTCCTGCGCTCTGGTTGCTGGGGCTCCAAATGCtaagaattcgagct
ENW_Tev_K3A	ggatcggatcctGAAAATTTATATTTTCAGAGCATGTCCCAGGAGAACCTGGAACAGTTTTACGTCCTCGTGCAAAACTCAGAACAATTACAAGAGCTGTTGGGCGCTACGGAAAACACAGATTCATTTAATGAACTCGCAGTGCGTTTAGGCCAGGATAATGGGTATAACTTTACCATCCAAGAAGTTGATGCTTTCGTCACCGAAAATCTGCAGAACGTTAACGCGGAACTGCGTGATGAAGAACTGGAACTGGTTGCGGGAGGTaaaGGAGCTTCGTGTCCGCTGGACACGCAATTCACGGCTTGCTTCCTGCGCTCTGGTTGCTGGGGCTCCAAATGCtaagaattcgagct

Supplementary Table S4: Accession numbers associated with proteins used in this study

Accession number	Protein
NpuA	WP_012412980.1
NpuM	WP_012412979.1
NpuN	WP_012412981.1

Supplementary Table S5: Strains associated with cluster 65 in the SSN (Fig. 1, main text) and their precursor peptides. Conserved residues that are predicted to be modified by class II lanthipeptide synthetases are highlighted.

Strain	Precursor sequence
Nostoc sp. NIES-2111	MNNSNLQNFYTLIQNSQELQAQLGAADSPESFAETAVRLGEENGYSFTTEDVNTFISQQRSRANAELSEADLEAVAGGKRRECPSDTRFTFCVFVSSCWGSAC
Nostoc punctiforme	MSQENLEQFYVLVQNSEQLQELLGATENTDSFNELAVRLGQDNGYNFTIQEVDAFVTENLQNVNAELRDEELELVAGGKGKSCPLDTQFTACFLRSGCWGSKC
Microcystis aeruginosa	MSQPDVERFYEIARNSEELQAMLGAAEDRDSFVETAVRLGQENGCNFTLDEANEFLNTKKGDAELSEQELEAVAGGRRRCNLNTRLTVCIIVSRNCWVATC
Nostoc sp. NIES-4103	MSKENLEQFYILVQNSEELQQQLGAIQDSQIFNETAVRLGQENGYSFTADEVDAFLNEKIQQNNAELSDRELEAVAGGKSRCPIGTRFTFCVLVSSCWGSKC
Mojavia pulchra JT2-VF2	MSKQNVEQFYIVVENNQQLQEQLGQADNPQSFYDMAARLGQENGYSFTAQEAEDFTNQRVQQGNAELSDQNLEAVAGGRSCPMDTRFTFCVFISSCWGSKC
Gloeotrichia echinulata HAB0833	MSKENLEQFYILVQNSEALQQQLGAIQESEVFNETAARLGQENGYSFSAEEVDTFLNEKKQQSNAELSDRELEAVAGGKGERCPINTRFTVCFLVSGCWGSRC
Aliinostoc sp. HNIBRCY26	MNNQNLYQFYTLIQNSQELQAQLGAAESPETFAETAVRLGEENGYSFTTEDVNAFVSQQASRANADLSEADLEAVAGGKAHTCPIDTRFTFCVFISECWGSVC
Nostoc sp.	MSKENLEQFYIFVENNQQLQEQLGQAENPQSFYEKAVQLGQENGYSFTVQEAEDFISQKAQQGNAELSDQQLEAVAGGKGGCPLDTKFTFCLLISSCLGSKC

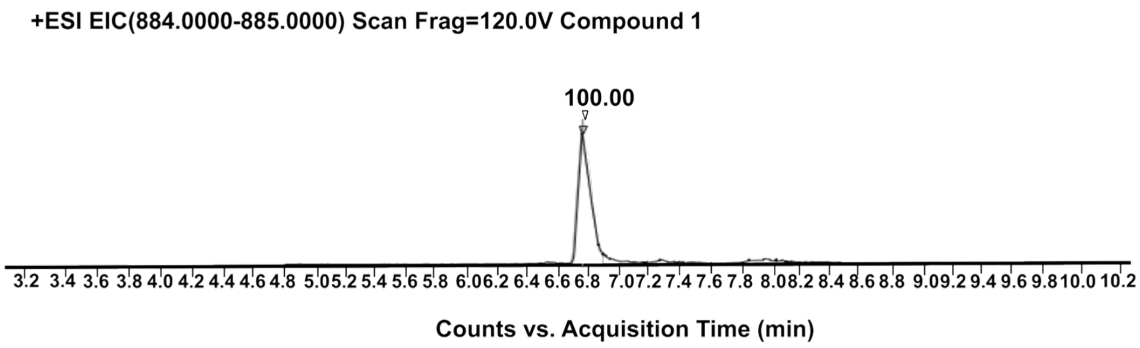

+ESI Scan (rt: 6.829 min) Frag=120.0V Compound 1

MH^{+1}(mono)	MH^{+2}(mono)	MH^{+3}(mono)	MH^{+4}(mono)	MH^{+5}(mono)
2651.2138	1326.1105	884.4095	663.5589	531.0486

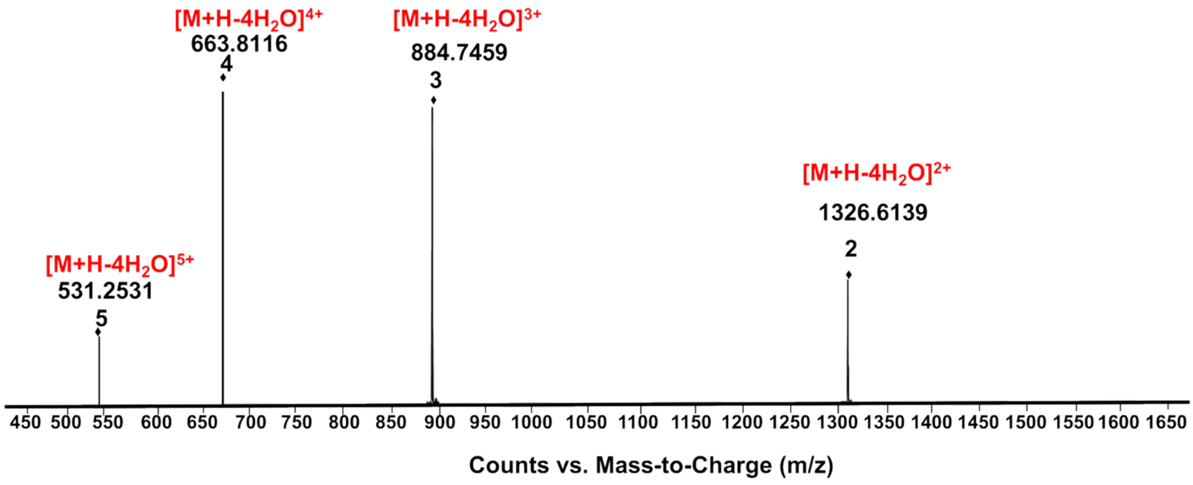

Figure S1. LC-QTOF-ESI/MS profile of nostolysamide C (positive mode) showing the monoisotopic mass for the different charge states for the 4-fold dehydrated product.

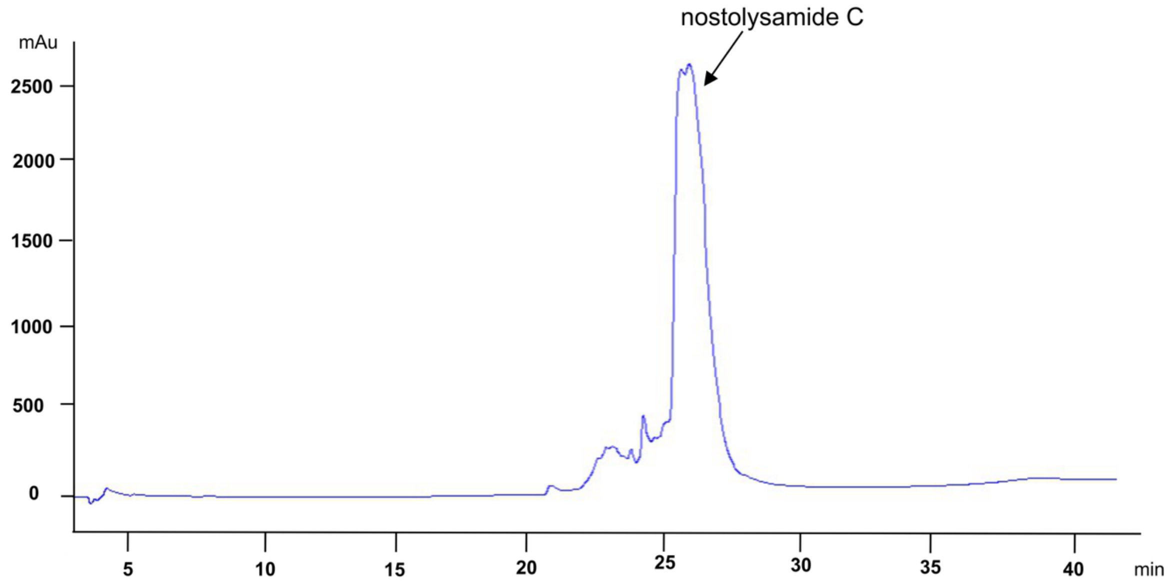

Figure S2. Reversed-phase HPLC trace of purified nostolysamide C (**1**), which elutes around 47% acetonitrile.

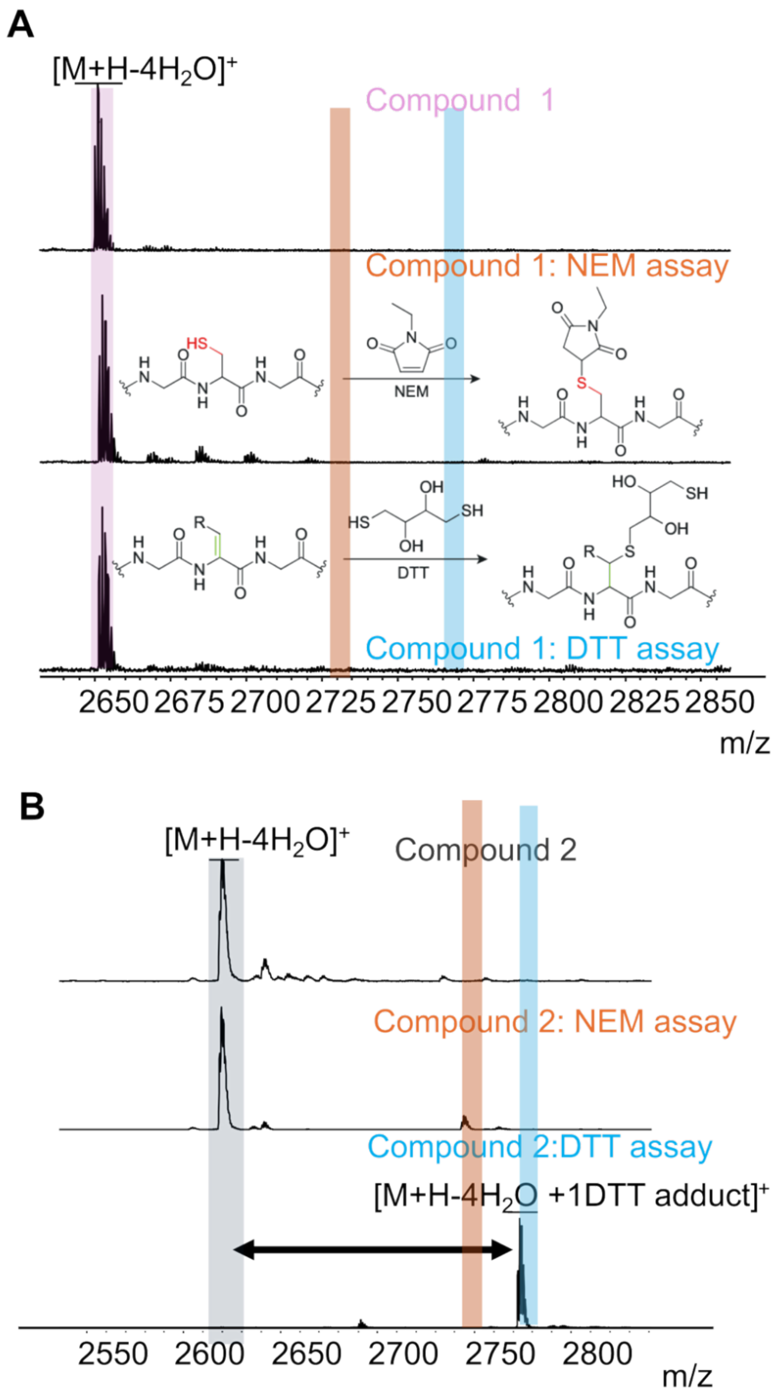

Figure S3. MALDI-TOF mass spectra of NEM and DTT assays.[1] (**A**) Compound **1** m/z: observed $[M+H-4H_2O]^+$=2651.3; calculated =2651.2. No adduct was observed when **1** was treated with NEM (expected m/z shown by red highlighted band) or DTT (expected m/z shown by blue highlighted band), indicating that the peptide is fully cyclized. (**B**) Compound **2** m/z: observed $[M+H-4H_2O]^+$= 2618.7; calculated= 2619.2. While no NEM adducts were formed showing the absence of a free Cys, a DTT adduct was observed, suggesting one dehydroamino acid in the product, as expected for this mutant. m/z observed $[M+H-4H_2O +1DTT\ adduct]^+$= 2772.5; calculated mass =2773.2.

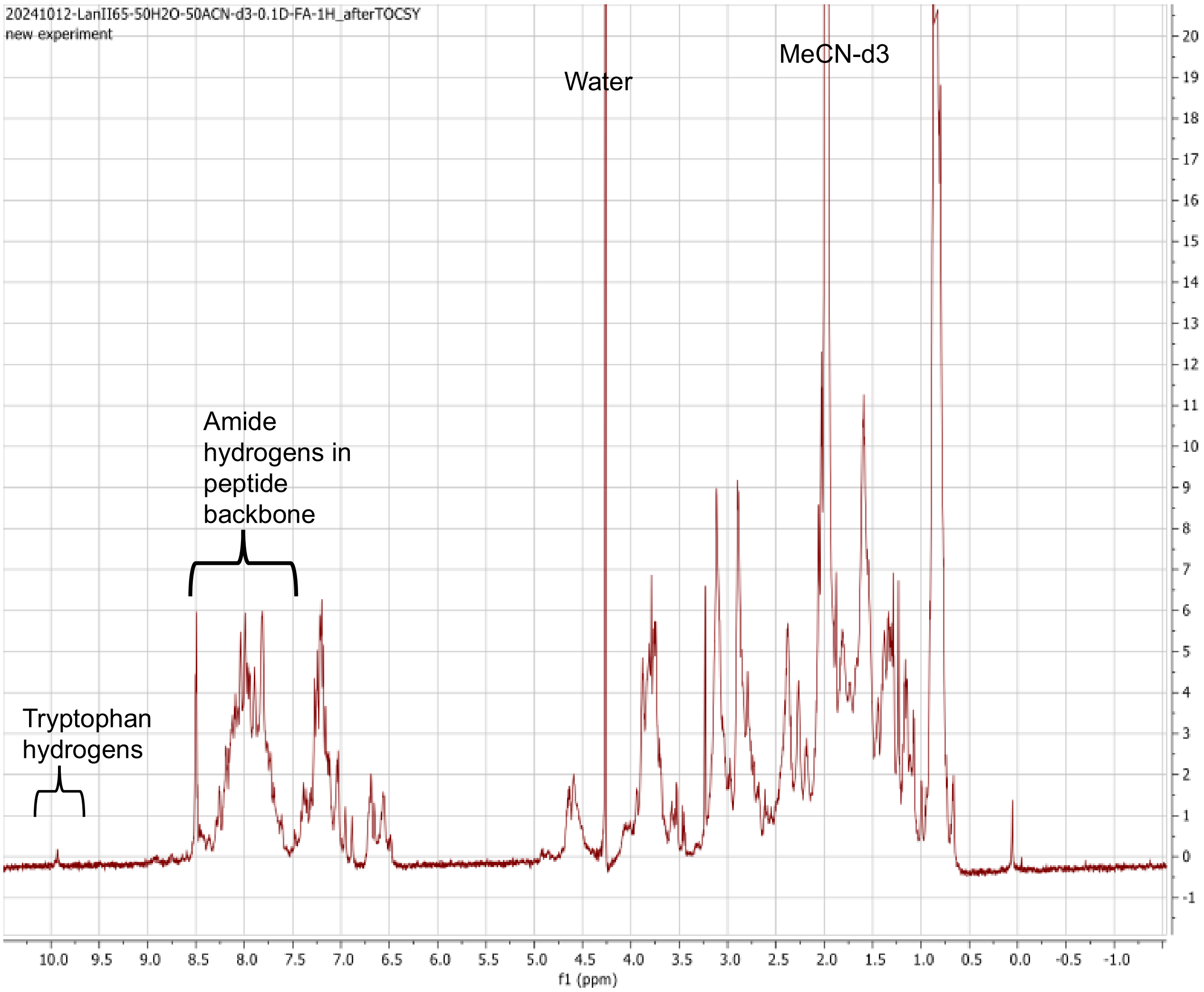

Figure S4: ^{1}H NMR spectrum of nostolysamide C (**1**) in 50% ACN-d_3/H_2O. The spectrum shows severe signal overlap in the amide region and poor signal dispersion. Furthermore, the solubility of the peptide was poor. Similar observations were made in other solvent systems.

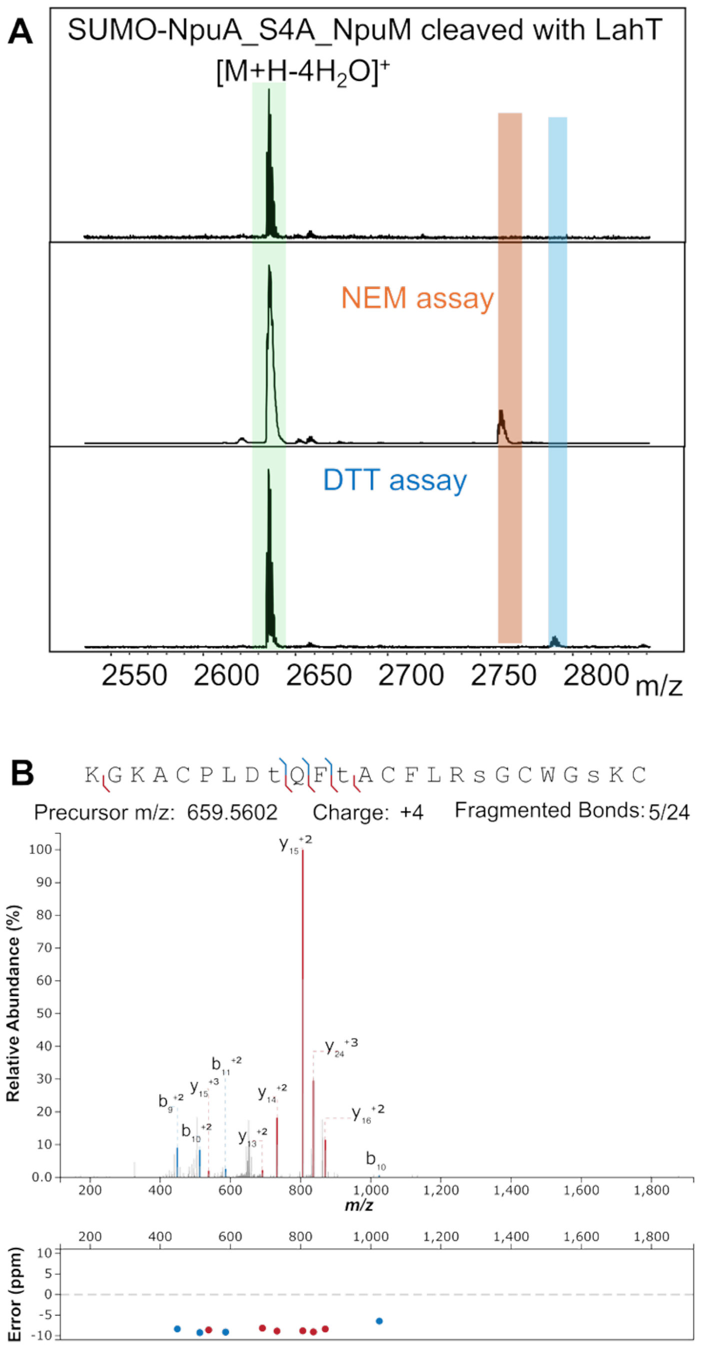

Figure S5. Characterization of SUMO-NpuA-S4A modified by NpuM in *E. coli* and cleaved in vitro with LahT150. (**A**) MALDI-TOF MS m/z observed [M+H-4 H_2O]$^+$=2634.3; calculated [M+H-4 H_2O]$^+$=2635.2. DTT and NEM assays showed no adducts, suggesting the serine at position 4 is not involved in the four dehydrations in WT NpuA. Furthermore, these assays show that the mutant peptide is fully dehydrated and mostly cyclized. (**B**) HR-MS of SUMO-NpuA-S4A modified by NpuM and cleaved with LahT150 showing b and y ions when the input sequence (lower-case letters) involved dehydrated residues at the indicated positions. Figure made using IPSA.[2] The fragmentation pattern is similar to wild-type NpuM-modified NpuA (**Fig. 2B**). HR-MS m/z calculated [M+H-4 H_2O]$^{4+}$= 659.5602 and observed [M+H-4 H_2O]$^{4+}$= 659.5602.

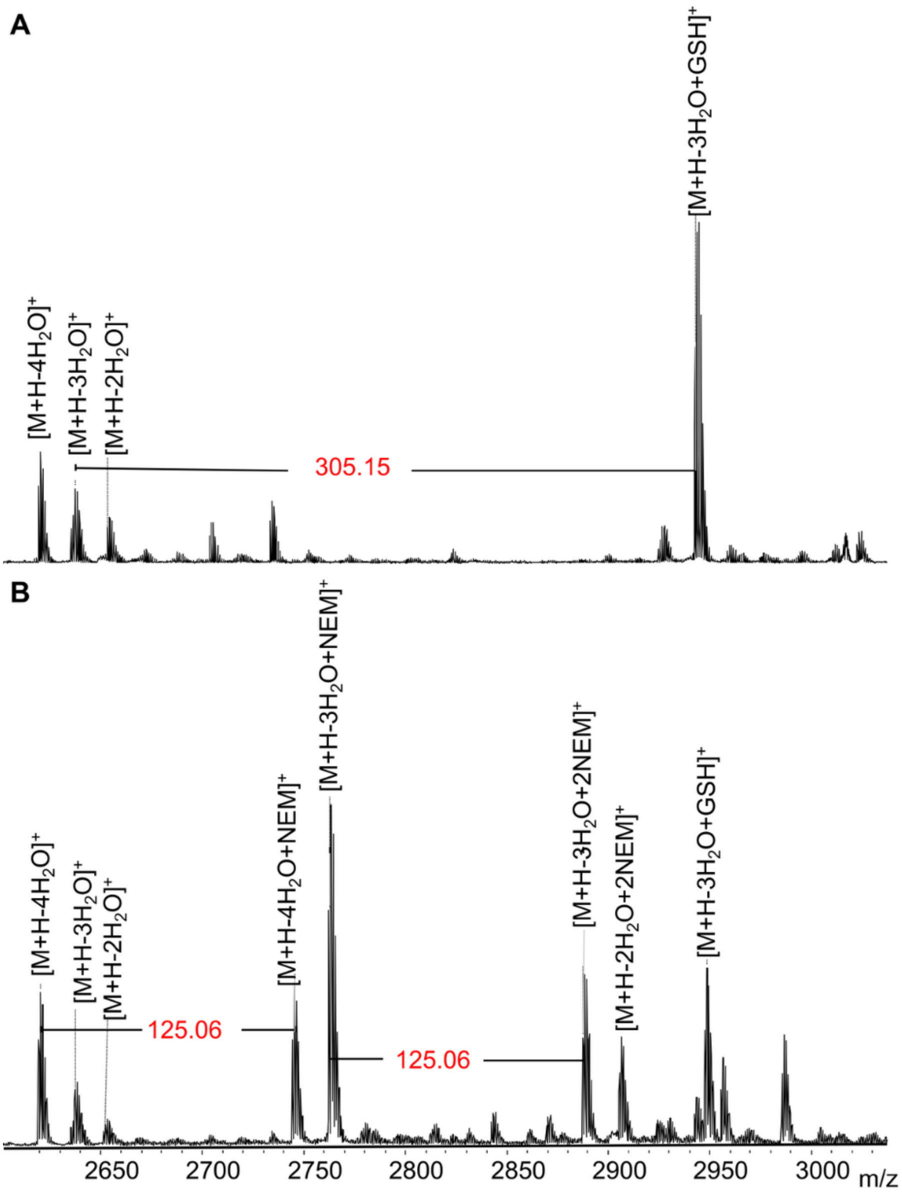

Figure S6. MALDI-TOF mass spectra of (A) NpuA-C14A co-expressed with NpuM, and cleaved with LahT150. MALDI-TOF MS showed a mixture of dehydrations, m/z observed $[M+H-4\ H_2O]^+$=2619.2, calculated $[M+H-4\ H_2O]^+$=2619.2; m/z observed $[M+H-3\ H_2O]^+$=2636.6, calculated $[M+H-3\ H_2O]^+$= 2637.2; m/z observed $[M+H-2\ H_2O]^+$=2653.5, calculated $[M+H-2\ H_2O]^+$= 2655.3 (loss of -2 Da likely because of disulfide bond formation). A glutathione adduct was observed on the three dehydrated products, resulting from a dehydroamino acid that was not cyclized with its partner Cys. (B) NEM assay showed several NEM adducts, which resulted in a +125 Da increase in m/z.

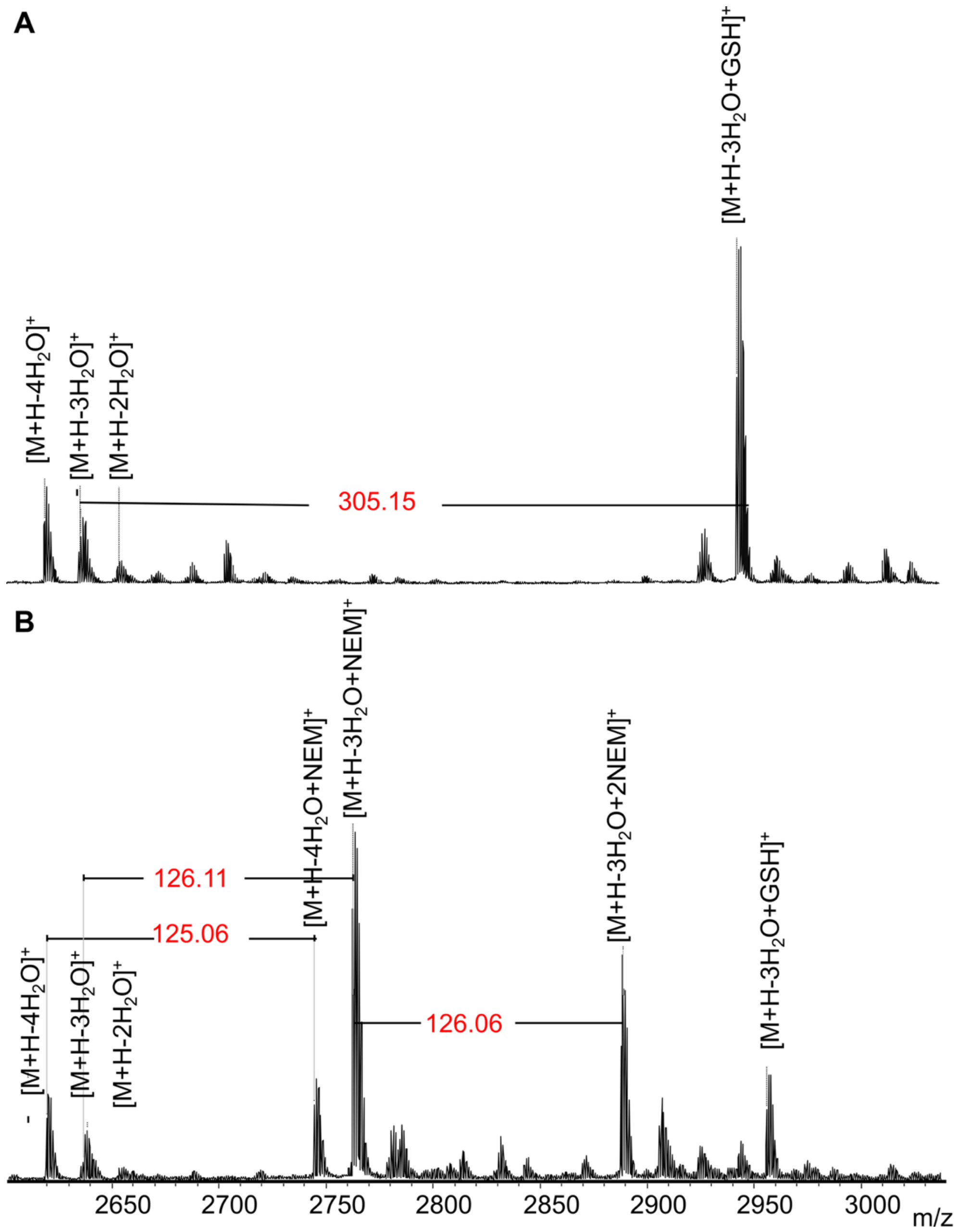

Figure S7. MALDI-TOF mass spectra of (A) NpuA-C20A co-expressed with NpuM, and cleaved with LahT150. MALDI-TOF MS showed a mixture of dehydrations, m/z observed [M+H-4 H_2O]$^+$=2619.5, calculated [M+H-4 H_2O]$^+$=2619.2; m/z observed [M+H-3 H_2O]$^+$=2636.5, calculated [M+H-3 H_2O]$^+$= 2637.2; m/z observed [M+H-2 H_2O]$^+$=2653.5, calculated [M+H-2 H_2O]$^+$= 2655.3 (loss of -2 Da likely because of disulfide bond formation). A glutathione adduct was observed on the three dehydrated products, resulting from a dehydroamino acid not being cyclized with its normal partner Cys. (B) NEM assay showed several NEM adducts, which resulted in a +125 Da increase.

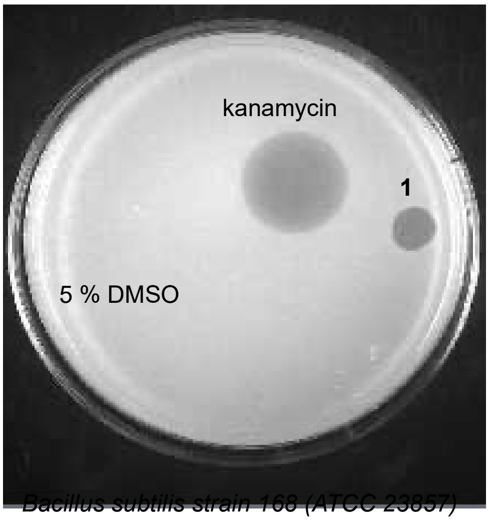

Figure S8. Bioactivity assay with nostolysamide C (1) against *B. subtilis*. A zone of bacterial growth inhibition was observed with *B. subtilis* 168 for both the positive control kanamycin (1 µL, 86 mM) and the tested compound **1** (2 µL,100 µM), while 5% DMSO (2 µL) served as a negative control.

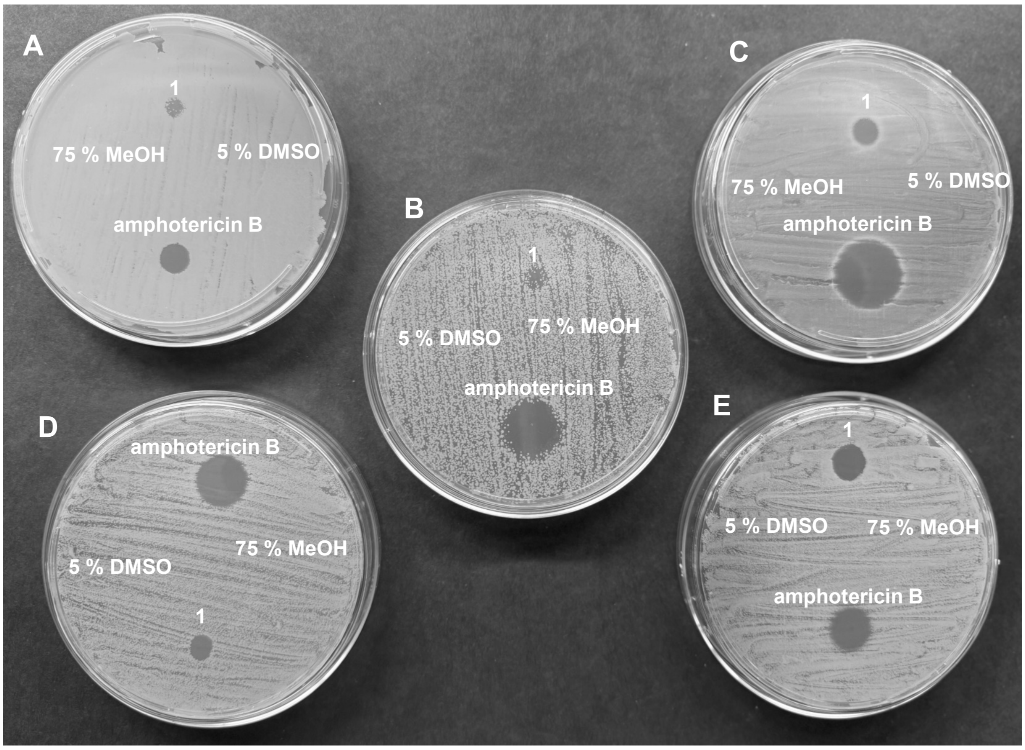

Figure S9. Bioactivity assay of nostolysamide C (1) against *Candida* species. Zones of growth inhibition were observed for compound **1** (2 µL, 400 µM) with *(*(**A**) *Candida krusei,* (**B**) *Candida albicans SN250*, (**C**) *Candida glabrata*, (**D**) *Candida albicans*, and (**E**) *Candida tropicalis.* Amphotericin B (2 µL, 1 mM) was used as the positive control and 5% DMSO (2 µL) and 75% MeOH (2 µL) served as negative controls.

A

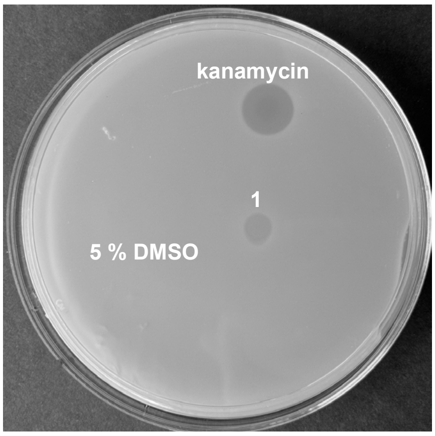

Escherichia coli

B

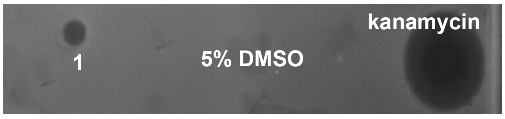

Klebsiella pneumoniae ATCC 27736

Figure S10. Bioactivity assay with nostolysamide C (1) against gram–negative bacteria. (**A**, **B**) Zones of bacterial growth inhibition were observed with *E. coli* (**A**) and *K. pneumoniae* (**B**) for the positive control kanamycin (2 µL, 0.86 mM) and compound **1** (2 µL, 200 µM); 5% DMSO (2 µL) was used as negative control.

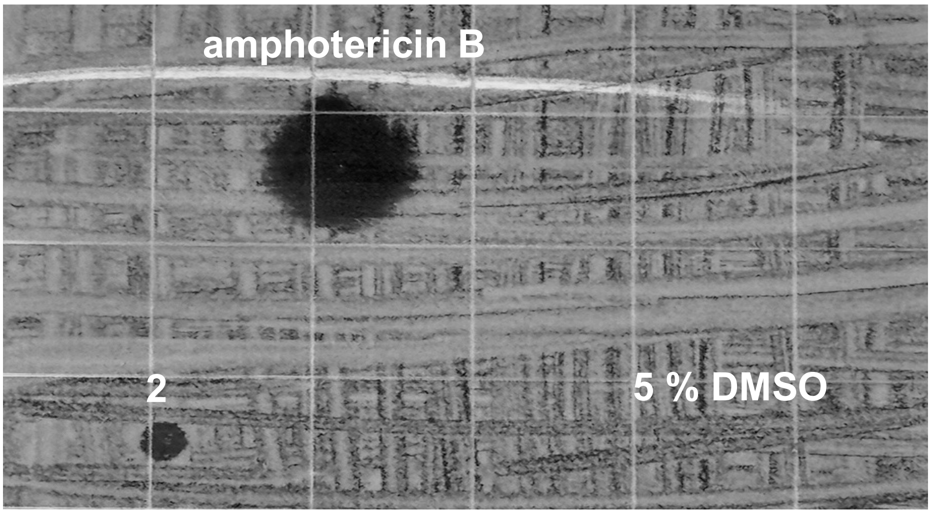

Figure S11. Bioactivity assay with nostolysamide C-C25A against *Candida tropicalis*. Zones of bacterial growth inhibition were observed against *C. tropicalis* with the positive control amphotericin B (2 µL, 1 mM) and compound **2** (2 µL, 200 µM); 5% DMSO (2 µL) served as the negative control.

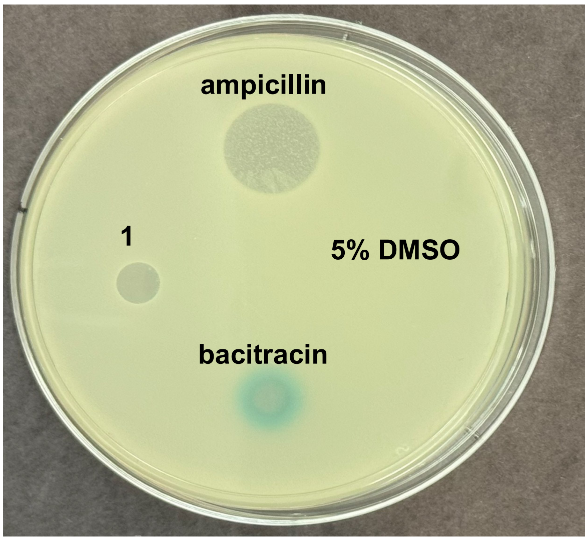

Bacillus subtilis BSF2470 (1A980)

Figure S12. To assess whether compound **1** targets lipid II, a reporter strain of *B. subtilis* was used with LacZ expression.[3] The soft agar contained X-gal. Amounts of compounds spotted were: 2 µL of 100 µM of compound **1**, 2 µL of 35 mM solution of the positive control bacitracin, and 2 µL of a 269 mM solution of the negative control ampicillin. As another control, 2 µL of 5% DMSO was also spotted.

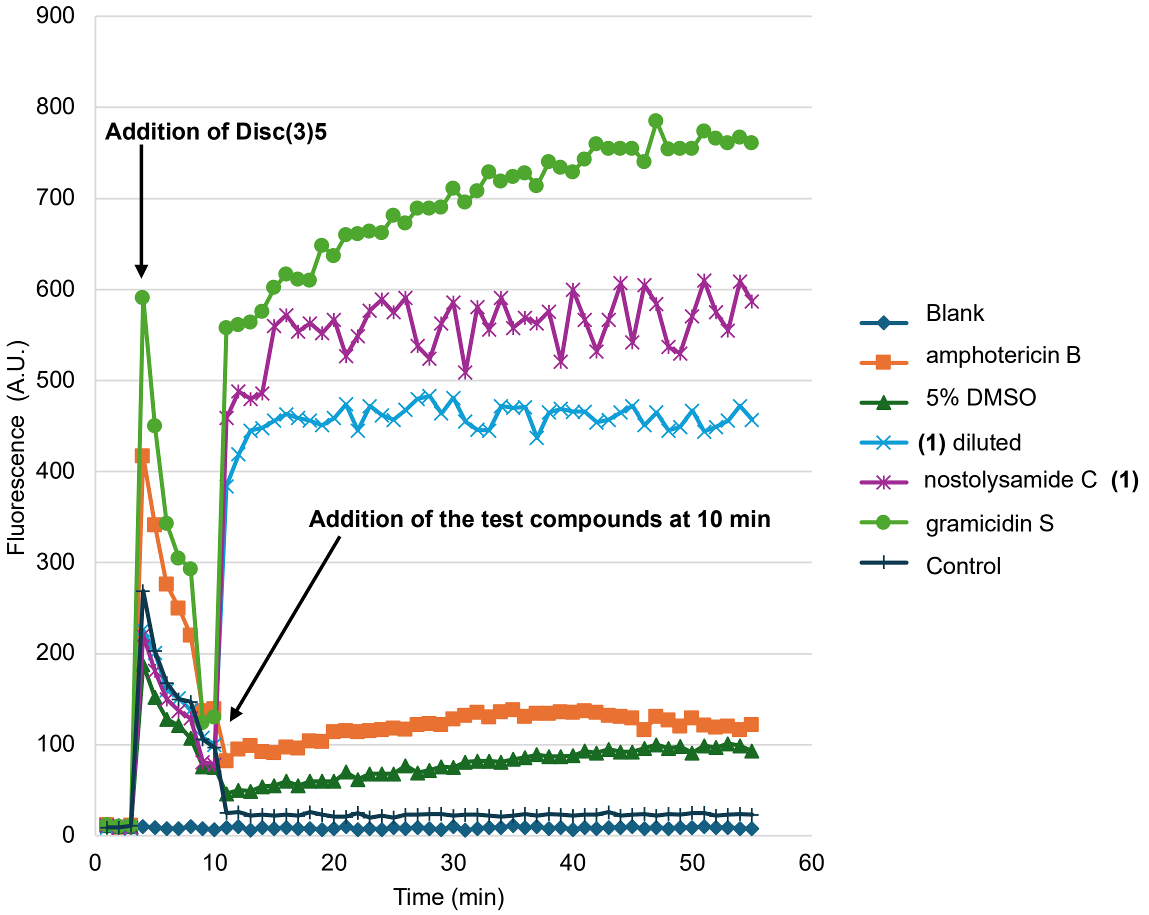

Figure S13. Membrane depolarization measured by DiSC$_3$(5) fluorescence. Fungal membrane potential was assessed using the voltage-sensitive dye DiSC$_3$(5). *Candida tropicalis* cells were incubated with DiSC$_3$(5) until fluorescence stabilized, followed by treatment with various test compounds/antibiotics. Increased fluorescence indicates dye release due to membrane depolarization. Gramicidin S (10 µL, 1 mM stock, 1 mM final) was used as the positive control. Nostolysamide C (**1**) (10 µL, 50-100 µM stock) showed similar fluorescence traces over time, suggesting nostolysamide C causes membrane depolarization.

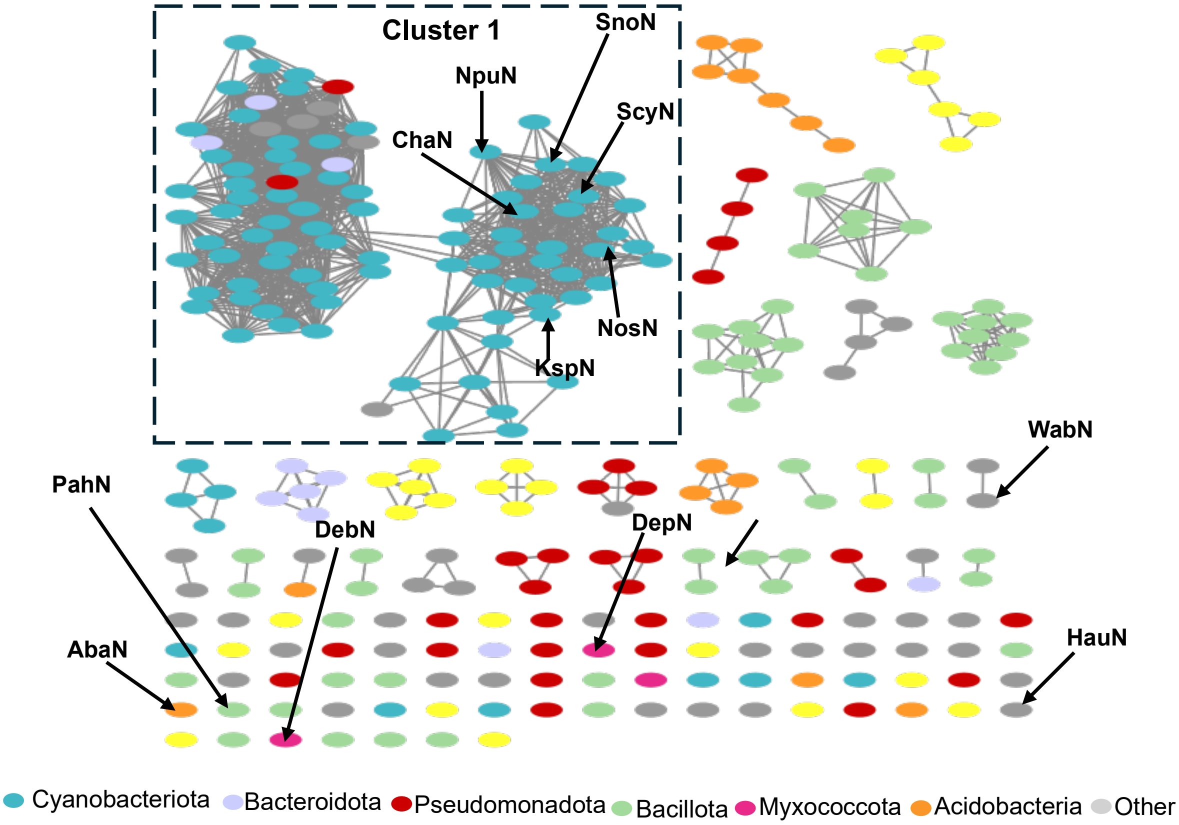

Figure S14. Sequence similarity network (SSN) of a subset of the GNAT-family acetyltransferases colored by the phyla they are encoded in. Protein sequences were obtained from UniProt via a BLAST search using NpuN as the query. The network was created by linking sequences sharing ≥ 70% pairwise identity. Nodes represent individual sequences, while edges indicate sequence similarity above this threshold. SSN construction and visualization were performed with the EFI-EST web tool with default parameters (minimum length 0, maximum length 50,000, E-value filter ≤100).[8] NpuN_repnode-1.00 is visualized in Cytoscape v3.10.[9] Highlighted is cluster 1, which includes NpuN and other multidomain acetyltransferases. Also indicated are other acetyltransferases identified by Piel and coworkers that are likely involved in RiPP biosynthesis.[10]

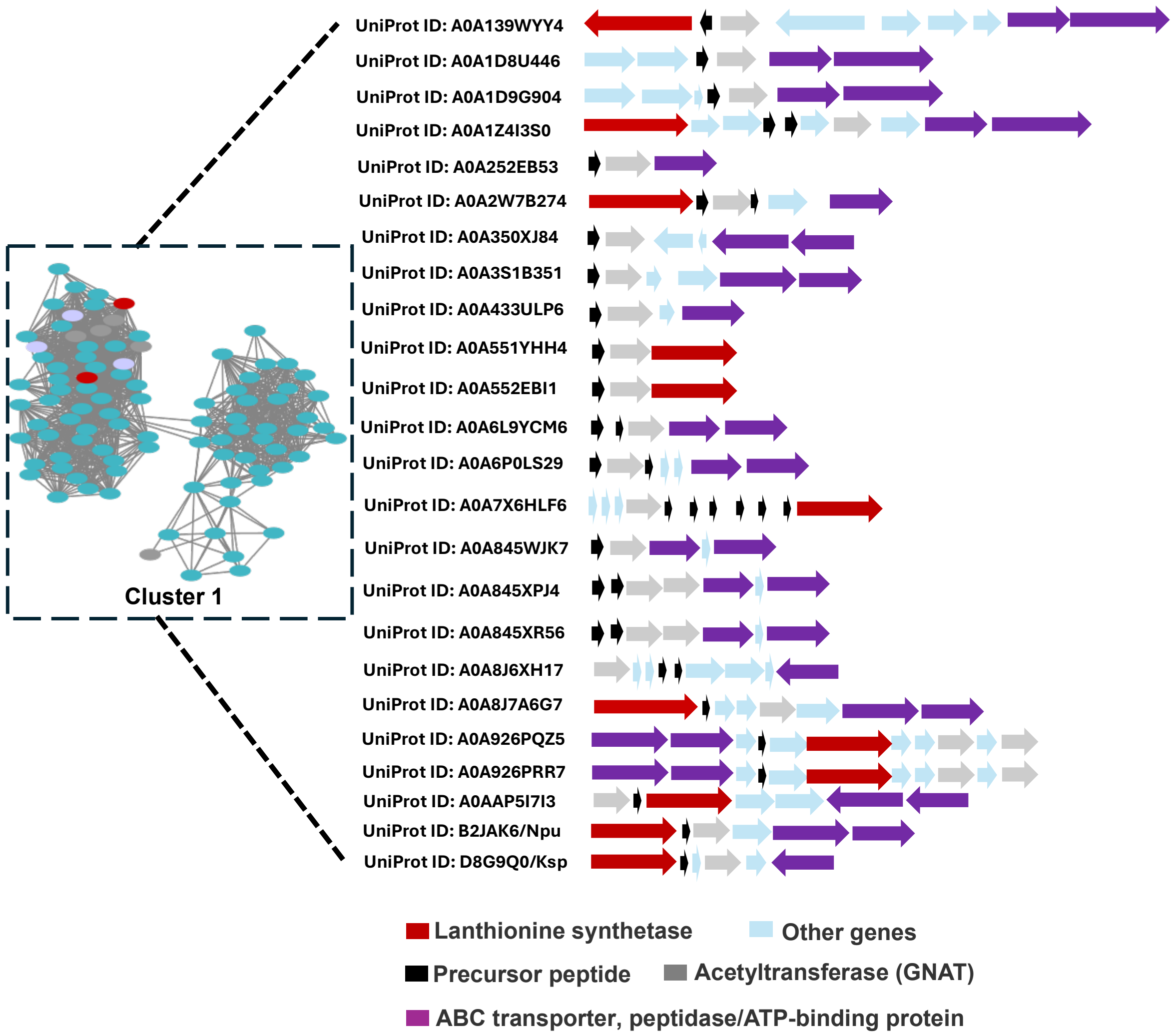

Figure S15. A representative subset of genome neighborhoods from Cluster 1 was identified using the Enzyme Function Initiative–Genome Neighborhood Tool (EFI-GNT). These neighborhoods were filtered to include BGCs encoding a conserved precursor peptide gene along with an acetyltransferase and/or lanthionine synthase. Coloring in the SSN is by phyla as in Fig. S14. Coloring of the BGCs is as shown in the legend of the Figure.

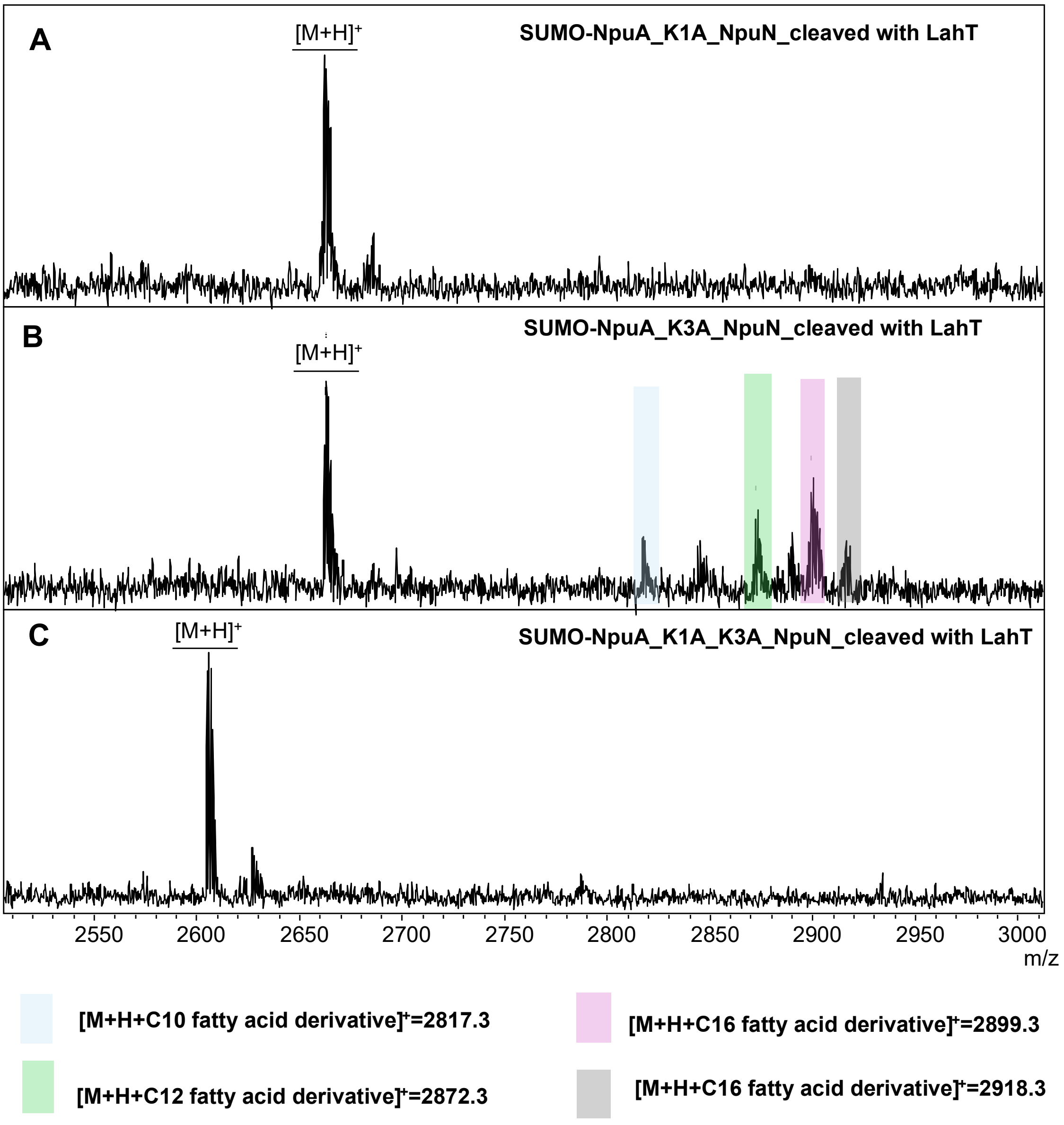

Figure S16. MALDI-TOF MS of the site-directed mutagenesis products of the lysine residues in the core peptide of NpuA to identify where acylation occurs. (**A**) SUMO-NpuA-K1A co-expressed with NpuN after cleavage with LahT150, m/z observed $[M+H]^+$= 2662.1, calculated 2666.2. No acylation was observed. (**B**) Lysine at position 3 of the core peptide of SUMO-NpuA was mutated to alanine and co-expressed with NpuN, resulting in products with different acyl groups as shown by MALDI-TOF MS. (**C**) Double mutation of the lysine residues at positions 1 and 3 of the core peptide of NpuA to alanine. No lipidation was observed.

A

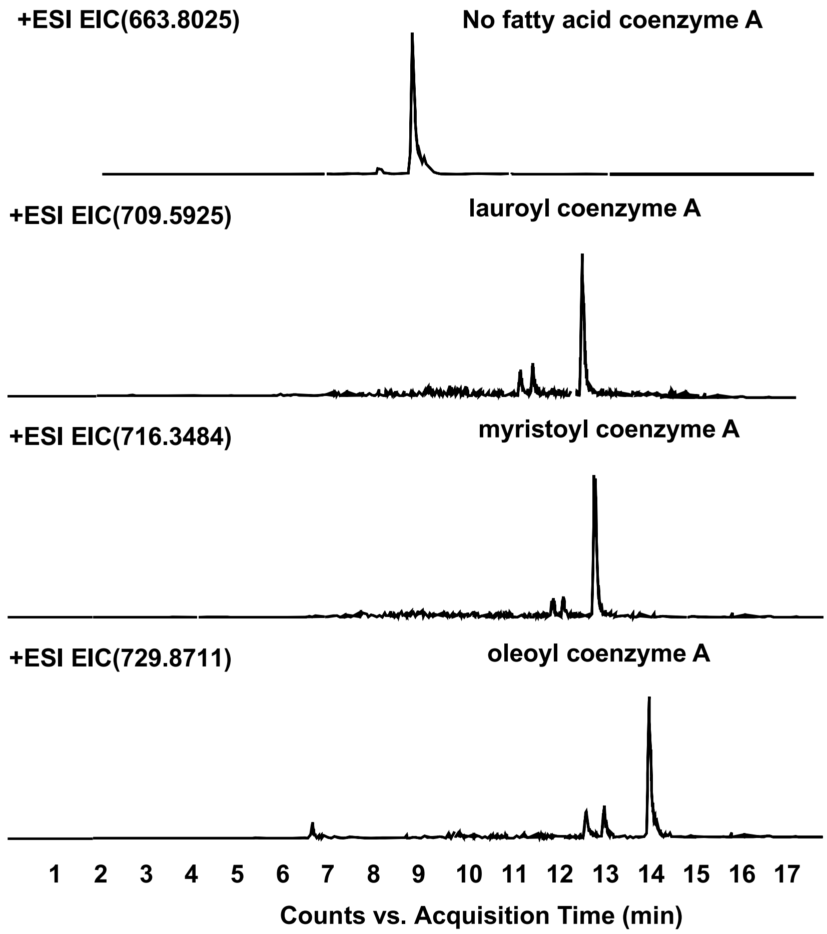

B

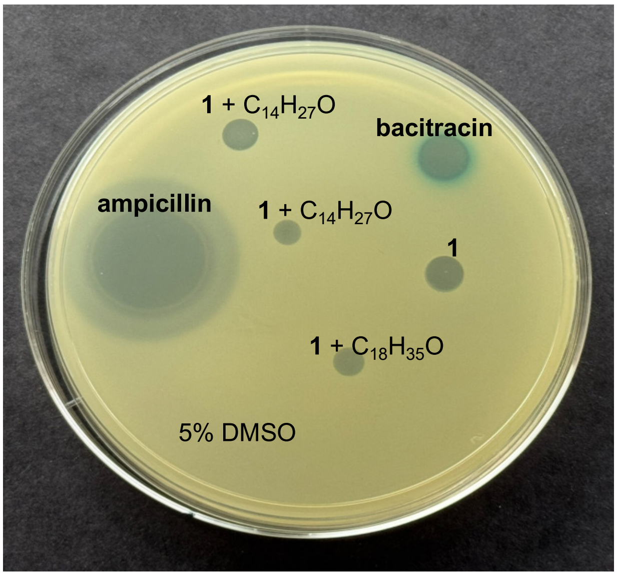

Bacillus subtilis BSF2470 (1A980)

Figure S17. (**A**). An agar diffusion assay was used to assess whether nostolysamide C and acylated variations target lipid II, using a reporter strain of *B. subtilis* with LacZ expression. The soft agar contained X-gal. Spotted were 2 µL of 100 µM of compound **1** and 2 µL of the same solution treated with NpuN and the indicated acyl-CoA (MS spectra shown in panel C).

Also spotted were 2 μL of a 35 mM solution of the positive control, bacitracin, and 2 μL of a 269 mM solution of the negative control, ampicillin. As another control, 2 μL of 5% DMSO was also spotted. (**B**) Extracted ion chromatograms (EICs) showing electrospray ionization MS analysis of the in vitro reactions of His-SUMO-NpuN (20 μL, 83 μM), nostolysamide C (20 μL, 250 μM, and different acyl-CoAs (10 μL, 1 mM).

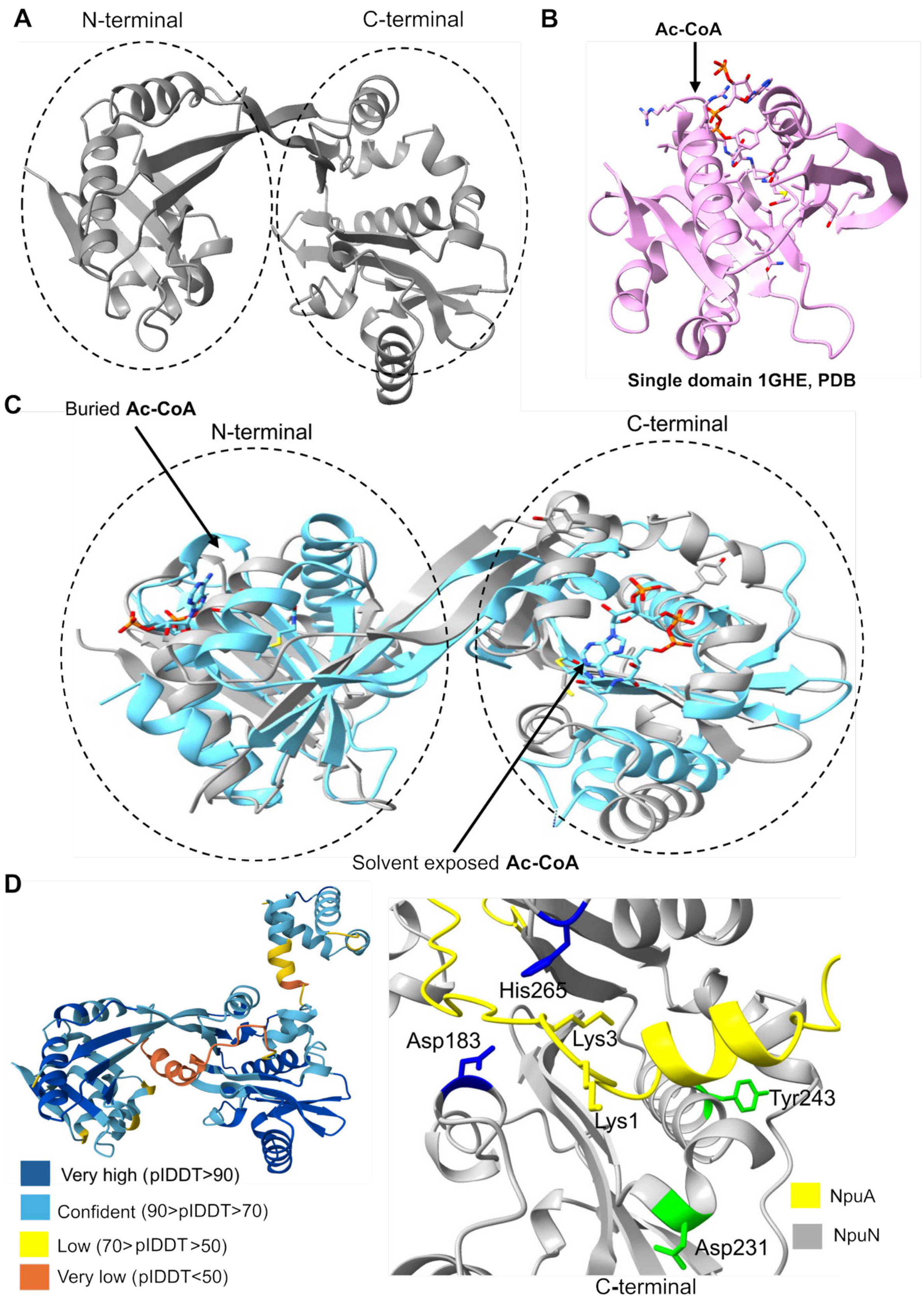

Figure S18. Structural comparison of characterized and AlphaFold-predicted[4] GNAT family proteins with single and tandem domain architectures. (**A**) AlphaFold3 model of NpuN, the tandem-domain GNAT enzyme investigated in this study, featuring two

acetyltransferase-like domains connected by a linker region. (**B**) The single-domain GNAT tabtoxin resistance (TTR) protein obtained from a DALI search displays the typical α/β acetyltransferase fold of the Gcn5-related family, with Ac-CoA docked (PDB ID 1GHE). (**C**) Structural alignment of two tandem GNAT proteins: grey for NpuN; blue for Virulence-induced protein F (VipF) from *Legionella hackeliae* with acetyl-CoA bound (PDB ID 6WQB).[5] The alignment illustrates how Ac-CoA might bind to the N- and C-terminal domains of NpuN, with one Ac-CoA buried vs one solvent exposed. (**D**) AlphaFold3 model of NpuN with the substrate NpuA colored by predicted Local Distance Difference Test (pLDDT) values and visualized using UCSF ChimeraX (left).[6] A close-up of the predicted C-terminal active site of NpuN with the substrate NpuA is shown on the right. Two residues, Asp183 and His265, in the C-terminal GNAT domain are positioned within approximately 5 Å of the substrate Lys1 ε-amino group that is acylated by NpuN, suggesting possible roles in acyl transfer. Conversely, a Tyr that has been implicated as general acid to protonate the thiolate leaving group in many GNATs[7], including VipF,[5] is conserved in NpuN (Fig. S19). However, this Tyr243 is not close to Lys1 of NpuA in the model of NpuN-NpuA. Thus, location of acyl-CoA and NpuA binding and the catalytic residues cannot be confidently predicted by the AlphaFold model.

A

```
NpuN      ---------------MYTL-------NILKDSTA--VMYEKMTFPAFQY-----------  25
TTR       MNHAQLRRVTAESFAHYRHGLAQLLFETVHGGASVGFMADLDMQQAYAWCDGLKADIAAG  60
                          *         : ::..::  .* :     *: :

NpuN      --VLQEINSKE-SLIAIAASYQEEPVGLVLAEIRTYNQSAEVLSLFVEPSHRCAGIGTNL  82
TTR       SLLLWVVAEDDNVLASAQLSLCQKPNGL---------NRAEVQKLMVLPSARGRGLGRQL  111
            :*  : ..:  * :   *  ::* **         : *** .*:* ** *  *:* :*

NpuN      LNRLETELTYRRCKKVELVYMTGKSTTPALENLLHKCGWTSPQPRMLVCKGEMEKGIQAP  142
TTR       MDEVEQVAVKHKRGLLHLDTEAGSVA----EAFYSALAYTR-------------------  148
          ::.:*   . ::   :.*   :*. :    * :    .:*

NpuN      WLKQYSQLPAAYTIFPWREITTQERQAIERQQATQPWIPEDLIPFKYDKQIEPLNSLGLR  202
TTR       ------------------------------------------------------------  148


NpuN      YQGQVVGWLIS--HRIFPDTIRYTCAFCRPDIQKKGRMITLFYEAAKLQLNAKIPHFIWT  260
TTR       -VGELPGYCATPDGRLHPTAIYF-KTLGQPT-----------------------------  177
            *:: *:  :   *:.* :* :  :: :*

NpuN      TPMNHQSMVAFVKYRCLPYLTSIQETRIISKLLLG   295
TTR       -----------------------------------   177
```

B

```
VipF      QGMIICNNQIDDDQLKAIQDLASLCREHDGGTPTFYNHLLIQKRPTENNVLYF---QDNQ  57
NpuN      ---MYTLNILKDSTAVMY--------EK-MTFPAF-QY-VLQEINSKESLIAIAASYQEE  46
NosN      ---MYELNILDNSTASAY--------EW-MTFPSY-KY-LLHTLDTDNTIVAIGATNNQQ  46
             :   * :.:.             *     *:: :: :::   :.:.:: :    :::

VipF      LLGFL--SVYFFYEDACEVSLIVSPLHRRQGIAKQLLQTIMPLLTAKEMTTLIFSTPTEI  115
NpuN      PVGLVLAEIRTYNQSAEVLSLFVEPSHRCAGIGTNLLNRLETELTYRRCKKVELVYMTGK  106
NosN      PVGLVLAQMVQ-DRIVVIFSIFVAIEERNKGIGTALLTRIQAELQDRGCKILQLNYTTGK  105
           :*::  .:    . .  .*::*   .*  **.. **  :   *  :  . : :   *

VipF      NDDWLINQGFSYRNSEYHMQRNGYDPIFMPTPKLHIRKATEDDIPALCAIDEACFPEHQE  175
NpuN      STT---------PALENLLHKCGWTS---PQPRMLVCKGEME-------------KGIQA  141
NosN      PTT---------AALERVIEKCNWTP---PETRTLVCKGEAK-------------RIIEA  140
                         *  :.:  .:     *  :  : *.  .                :

VipF      NMISRFSMLLNDASYTLFLASY------NHIIVGKAHIHWQSKEAIF---SDIAIFRQYQ  226
NpuN      PWLKQYSQLP--AAYTIFPWREITTQERQAIERQQATQPWIPEDLIPFKYDKQIEPLNSL  199
NosN      NWIRRYSRLP--SSYSIFPWEEITPEERQIIQQQQQTQPWIPEDLVPFKYEDNLEPLNSI  198
            : ::* *   ::*::*          : *   :    *  :: :    ..     :

VipF      GQGWGGELLSYCINQALTSGKNKLML-----DVETSNQNALHLYT---RLGFKTANVSDY  278
NpuN      GLRYQGQVVGWLISHRIFPDTIRYTCAFCRPDIQKKG-RMITLFYEAAKLQLNAKIPHFI  258
NosN      GLRYQGQVVGWLINHRIAPHTIRYTCSFVREDLQKMG-RIIILYAEACERQIKANIPNAI  257
          *  : *:::.: *.: :   . :        *::. . . : *:    .  :::

VipF      WVIPLPQLLT------NWALE--------------- 293
NpuN      WTTPMNHQSMVAFVKYRCLPYLTSIQETRIISKLLLG 295
NosN      WTVPSFHQSMVSFVKNHWLPYLNAVEESRGTLKLI-- 292
          *. *  :         .
```

Figure S19. (**A**) Sequence alignment of the N-terminal domain of NpuN and TTR. The two proteins display 18.83% sequence identity. Highlighted in the pink box is the P-loop motif

Q/R-X-X-G-X-G/A, which anchors acetyl-CoA in TTR and VipF. This sequence motif was observed in NpuN at the N-terminus. (**B**) Multiple sequence alignment of tandem GNAT protein VipF with NpuN and NosN (see also Fig. S18). The P-loop motif is boxed solid in the N-terminal domains, and dashed in the C-terminal domains. Unlike VipF that has two canonical P-loops, NpuN and NosN have atypical P-loop motifs at the C-termini with an Asn replacing the Gln and an Arg replacing the second Gly. Two conserved residues in NpuN and NosN that are close to Lys1 of NpuA in an AlphaFold3 model of the complex (Fig. S18D) are highlighted in blue. Two other residues that have been implicated in catalysis in VipF[5] are highlighted in green. The sequence identity between VipF and NpuN is 25.1%, whereas NpuN and NosN have a sequence identity of 55.3%.

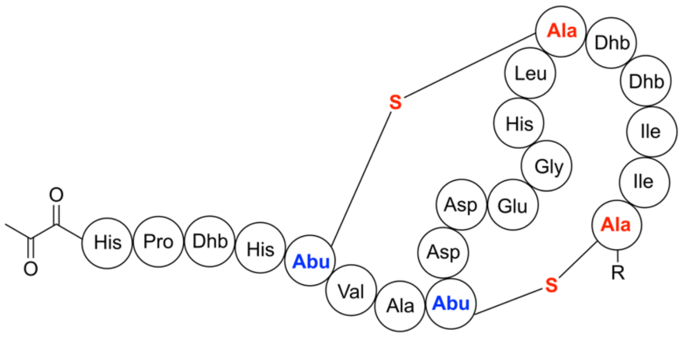

Pinensin A, R = Ala-OH
Pinensin B, R = OH

Figure S20. Chemical structures of pinensins A and B.[11]

```
NpuM   870  KFDIIGGAAGAILGLLALAQEQPMVLKQAIACGQHLLKYCNE-VFGKTSNL
ProcM  807  QLDLIGGCAGLIGSLVQLGTES-ALQWALRAGDHLIAQQNEEGAWSSSSSQ

NpuM   920  KGLIGFSHGAAGIAYALLRLYAVTQDTDYLDAARMAIAYENALFYPSVGNWR
ProcM  857  PALLGFSHGVAGYAAALAHLHAFSGDERYRIAAAAALAYERARFNKDAGNWP

NpuM   972  EITPVY--DPASLPVFWSTWCHGAPGIALGRLGSLSIDR-SEQILTDIEVA
ProcM  909  DYRSISRDSDSDEPSFMASWCHGAPGIALGRACLWGTALWDEECTKEIGIG

NpuM  1020  LKTTQNTALQTIDHLCCGNLG
ProcM  960  LQTTAAVSAVSTDHLCCGSLG
```

Figure S21. Sequence alignment of the C-termini of NpuM and ProcM. The Cys residues involved in Zn^{2+} coordination in the active site are shown in red.

References

1. R. S. Ayikpoe and W. A. van der Donk, *Methods Enzymol.*, 2021, **656**, 573-621.
2. D. R. Brademan, N. M. Riley, N. W. Kwiecien and J. J. Coon, *Mol. Cell. Proteom.*, 2019, **18**, S193-S201.
3. T. Mascher, S. L. Zimmer, T. A. Smith and J. D. Helmann, *Antimicrob. Agents Chemother.*, 2004, **48**, 2888-2896.
4. J. Jumper, R. Evans, A. Pritzel, T. Green, M. Figurnov, O. Ronneberger, K. Tunyasuvunakool, R. Bates, A. Žídek, A. Potapenko, A. Bridgland, C. Meyer, S. A. A. Kohl, A. J. Ballard, A. Cowie, B. Romera-Paredes, S. Nikolov, R. Jain, J. Adler, T. Back, S. Petersen, D. Reiman, E. Clancy, M. Zielinski, M. Steinegger, M. Pacholska, T. Berghammer, S. Bodenstein, D. Silver, O. Vinyals, A. W. Senior, K. Kavukcuoglu, P. Kohli and D. Hassabis, *Nature*, 2021, **596**, 583-589.
5. L. Syriste, D. T. Patel, P. J. Stogios, T. Skarina, D. Patel and A. Savchenko, *mBio*, 2024, **15**, e03221-03223.
6. E. C. Meng, T. D. Goddard, E. F. Pettersen, G. S. Couch, Z. J. Pearson, J. H. Morris and T. E. Ferrin, *Protein Sci.*, 2023, **32**, e4792.
7. M. W. Vetting, S. d. C. LP, M. Yu, S. S. Hegde, S. Magnet, S. L. Roderick and J. S. Blanchard, *Arch. Biochem. Biophys.*, 2005, **433**, 212-226.
8. N. Oberg, R. Zallot and J. A. Gerlt, *J. Mol. Biol.*, 2023, **435**, 168018.
9. P. Shannon, A. Markiel, O. Ozier, N. S. Baliga, J. T. Wang, D. Ramage, N. Amin, B. Schwikowski and T. Ideker, *Genome Res.*, 2003, **13**, 2498-2504.
10. F. Hubrich, N. M. Bösch, C. Chepkirui, B. I. Morinaka, M. Rust, M. Gugger, S. L. Robinson, A. L. Vagstad and J. Piel, *Proc. Natl. Acad. Sci. U S A*, 2022, **119**, e2113120119.
11. K. I. Mohr, C. Volz, R. Jansen, V. Wray, J. Hoffmann, S. Bernecker, J. Wink, K. Gerth, M. Stadler and R. Muller, *Angew Chem Int Ed Engl*, 2015, **54**, 11254-11258.
